# The Effects of Same- and Other-Race Facial Expressions of Pain on Temporal Perception

**DOI:** 10.3389/fpsyg.2018.02366

**Published:** 2018-11-29

**Authors:** Shunhang Huang, Junjie Qiu, Peiduo Liu, Qingqing Li, Xiting Huang

**Affiliations:** ^1^Faculty of Psychology, Southwest University, Chongqing, China; ^2^The School of Educational Science, Lingnan Normal University, Zhanjiang, China

**Keywords:** time perception, facial expressions of pain, race, arousal, attention

## Abstract

Previous studies suggested that threatening stimuli lengthen subjective duration, while facial expressions of pain were found to produce a shortening effect on temporal perception in a recent study. Moreover, individuals’ responses to others’ pain were influenced by the individuals’ relationship to a racial group. However, the effects of same- and other-race pained facial expressions on temporal perception, remain unknown. The aim of this present study was to identify the effect expressions of pain have on temporal perception and to explore whether this effect was modulated by the relationship to a racial group. In a temporal bisection task, Chinese participants were presented with pain or neutral facial expressions displayed by Caucasian (other-race) or Chinese (same-race) models in a 400–1600 ms or 200–800 ms condition. Expressions of pain were rated as more arousing, negative and disagreeable, than neutral facial expressions. These scores were not significantly different between same- and other-race facial expressions. Based on the results of the temporal bisection task, both same- and other-race pained facial expressions lengthened the perceived duration in the 400–1600 ms condition, but only same-race pained facial expressions produced this effect in the 200–800 ms condition. We postulate that the existence of a short-lived effect of pained facial expressions on lengthening temporal perception caused by arousal and attention, occurs at an earlier time point for same-race pained facial expressions than for other-race pained facial expressions.

## Introduction

Accurate perceptions of time are crucial in daily life. However, according to numerous empirical findings, a subjective duration is distorted by internal and external factors, and one particularly significant factor is emotion. Various emotional stimuli, such as affective images selected from the International Affective Picture System (IAPS) (Balsam and Van Volkinburg, 2014; Tamm et al., 2014) and emotional facial expressions (Gil and Droit-Volet, 2011; Ng et al., 2011; Grondin et al., 2014), have been employed to explore the underlying mechanisms of the effects of emotion on a subjective time experience. A relatively consistent finding was that threatening stimuli with high arousal and negative valence extends an observers’ subjective duration. This temporal lengthening effect may help individuals to efficiently and adaptively respond to occurring or forthcoming stimuli (Droit-Volet and Gil, 2009; Lake et al., 2016).

Painful stimuli can lengthen the perceived duration, which is of great adaptive value. When an organism is damaged, the experience of pain warns the organism of a threat and induces behaviors to eliminate, weaken or escape the source of the pain (Bonica, 1987; Jamal et al., 2003). Consistent with the hypothesis that threatening stimuli lengthen the subjective duration, previous studies have found that a physically painful stimulus lengthens the subjective duration from hundreds of milliseconds to a few seconds. For example, when participants’ hands are immersed in painfully cold water, participants tend to overestimate the duration in a temporal bisection task compared to immersion in water with a neutral temperature (Rey et al., 2017). Therefore, the lengthening effect of one’s own physical pain on temporal perception was found to be relatively stable.

Facial expressions are important in daily social communication (Ekman, 1993; Coulson et al., 2004). The facial expression of pain, which is distinct and distinguishable from other facial expressions of basic emotions, can signal threats to others to obtain caregiving and help. This process is critical for the sufferer and is ultimately beneficial for the survival of the entire group (Williams, 2002; Simon et al., 2008). Similarities exist between the perception of one’s own and others’ pain. As in nociception, when one witnesses another’s expression of pain, the brain regions known as the pain matrix, including the anterior cingulate cortex and anterior insula, are activated (Simon et al., 2006; Xu et al., 2009; Cui et al., 2015). Therefore, facial expressions of pain also conceivably prolong subjective duration, analogous to the effect of the direct exposure to pain. However, a recent study, in which participants were presented with facial expressions of pain, found that the stimuli shortened the time perception (Ballotta et al., 2018). As the emotional effect on temporal perception was sensitive to the tasks and the durations used (Gil and Droit-Volet, 2011, 2012; Lake et al., 2016), a dubious conclusion is that another individual’s pain produces a shortening effect on temporal perception. More studies are needed to identify the effects of facial expressions of pain.

Interestingly, individuals’ neural responses to others’ pain are influenced by the racial group relationships between the sufferer and the observer. According to recent event-related brain potential (ERP) studies, the neural response to pain expressed by same- and other-race individuals relies on different processes. For example, increased neural responses were observed in the frontal/central brain regions at 128–188 ms (P2) and 200–300 ms (N2) after a stimulus onset when Chinese participants viewed facial expressions of pain compared to neutral expressions displayed by same-race models. However, this effect was not significant when other-race facial expressions were used as the stimuli. The P2 effect was positively correlated with the unpleasant feelings induced by a pain perception and reflected an early automatic neural response to pain (Sheng and Han, 2012; Sheng et al., 2013; Huang and Han, 2014). Similarly, an early race-biased stage of pain sharing at approximately 280–340 ms and a later race-unbiased stage regarding pain cognition at approximately 400–750 ms were identified when Caucasian participants were presented with Caucasian and African models receiving painful stimuli (Sessa et al., 2014). Accordingly, the processing of same- and other-race facial expressions of pain exhibited differing temporal courses; the pain of same-race members was perceived more quickly and automatically than the pain of other-race members.

The mechanisms by which the facial expressions of same- and different-race individuals affect temporal perception are poorly understood. To our knowledge, only one study has investigated the impact of same- and other-race facial expressions of emotion on time perception. Caucasian participants overestimated the duration of the presentation of angry facial expressions, compared to the duration for neutral facial expressions, but this effect was only significant for in-group facial expressions. However, the lengthening effect of facial expressions of anger on Chinese participants was evident for both same-race and other-race facial expressions (Mondillon et al., 2007). Therefore, the modulatory effect of the racial group relationship on emotional timing is complicated, and further data are needed. Studies on the effects of same- and other-race facial expressions of pain, on time perception may be useful to improve our knowledge of this issue.

The internal clock theory suggests that facial expressions of pain may modulate temporal perception through arousal and/or attention. According to the scalar expectancy theory (SET) (Gibbon, 1977; Gibbon et al., 1984), arousal and attention are two main factors that account for emotion-induced temporal distortion (Gil and Droit-Volet, 2012; Lake, 2016; Tipples et al., 2016). Because others’ pain may spur the observer to react quickly to threatening stimuli through heightened arousal and attention (Williams, 2002; Lamm et al., 2007), the SET may be useful to elucidate the effect of facial expressions of pain, on temporal perception. Within the SET framework, which is composed of a pacemaker, an accumulator and a switch, the rate of the pacemaker accelerates when the level of arousal increases, causing the pacemaker to emit more pulses within a specific duration and ultimately resulting in a longer perceived duration (Gil and Droit-Volet, 2012; Tipples et al., 2016). For example, compared to emotions with lower arousal levels (sadness and happiness), fearful and angry expressions with higher arousal levels cause longer perceived durations (Droit-Volet et al., 2004). Importantly, arousal-induced changes in the rate of the pacemaker produce a multiplicative effect on the subjective duration. Attention also lengthens the perceived duration, and its effect differs from arousal; when the switch opens earlier or closes later, more units are collected by the accumulator. The latencies of open and closed states are independent of the temporal duration timed; thus, the effect of attention on temporal perception is additive rather than multiplicative (Meck and Benson, 2002). The mathematical distinction between the effects of attention and arousal on time perception may help us to understand the effects of same- and other-race facial expressions of pain, on temporal perception by employing different temporal durations (Droit-Volet et al., 2010; Gil and Droit-Volet, 2012).

The aim of the current study is to explore the effect of facial expressions of pain on temporal perception and whether this effect is modulated by the racial group relationship. We employed the temporal bisection task because it activates the core timing network (Tipples et al., 2013) and is sensitive to the effects of emotional stimulus on temporal perception (e.g., Gil et al., 2009; Fayolle et al., 2015; Droit-Volet et al., 2016). In this task, participants are trained to remember two different standard durations as the short and long standard durations. The participants are then presented with facial expressions of pain or neutral expressions displayed by Chinese or Caucasian models; these facial expressions are presented for seven different durations that fall between the two learned times and are separated into two blocks based on race. The participants are asked to indicate whether the time period of the present stimulus was closer to the “short” or the “long” standard duration. In Experiment 1, we adopted for 400 and 1600 ms as the short and the long standard durations, respectively; these durations have been used extensively in previous emotional time-perception studies (e.g., Droit-Volet et al., 2010; Grommet et al., 2011; Tipples et al., 2013). We used durations ranging from 200 to 800 ms in Experiment 2, to test whether the lengthening effect of facial expressions of pain was multiplicative or additive. These durations were also adopted in previous studies (e.g., Gil and Droit-Volet, 2012; Fayolle et al., 2014; Droit-Volet and Gil, 2016). The present study hypothesized that facial expressions of pain would produce a lengthening effect on temporal perception and that the lengthening effect produced by the facial expressions of pain of same-race members, would be larger than that of other-race members.

## Experiment 1

### Materials and Methods

#### Participants

An a priori power analysis was adopted using G^∗^Power software to determine the sample size (Faul et al., 2007). The effect size was set to 0.4 and the alpha was set to 0.05. The results indicated that 21 participants were sufficient.

Thirty-eight right-handed Chinese students (10 males and 28 females) from Southwest University (SWU) participated in the study (mean age = 19.89, *SD* = 2.04). All participants had normal or corrected-to-normal vision. This study was approved by the SWU Research Ethics Committee.

#### Materials and Procedure

Each participant was tested individually in front of a computer screen. E-prime software (1.2, Psychology Software Tools, Pittsburgh, PA, United States) was installed on computers and used to control the experimental process. The participants were instructed to press one of two keys (“K” or “D”) to respond. The stimuli participants needed to estimate were a brown rectangle (6.84° × 9.22°) in the training phase and 40 digital color static images (6.84° × 9.22°) of facial expressions of pain or neutral facial expressions, displayed by 10 Caucasian models (5 males) and 10 Chinese models (5 males) that were selected from previous studies (e.g., Sheng and Han, 2012) in the test phase.

Similar to previous studies (Simon et al., 2008; Sheng and Han, 2012), after the temporal bisection task, each participant was required to rate their own unpleasant feelings and arousal for each facial expression using a 9-point Likert scale (1 = not at all unpleasant or extremely calm; 9 = extremely unpleasant or excited). In addition, because facial attractiveness affects temporal perception (Ogden, 2013), participants were instructed to assess how likable they found each face using a 9-point Likert scale (1 = not at all; 9 = extremely strong).

These scores were subjected to a 2 (models’ race: Chinese vs. Caucasian) × 2 (facial expressions: pain vs. neutral) analysis of variance (ANOVA). As expected, lower scores were recorded for self-unpleasantness and arousal for neutral facial expressions than for pained facial expressions [*F*(1,37) = 162.51 and 144.1, all *p* < 0.01, $\eta_{p}^{2}$ = 0.82 and 0.80]. However, a significant racial effect was not observed [*F*(1,37) = 0.74 and 0.03, all *p* > 0.1, Table 1]. The likeability scores were lower for facial expressions of pain than for neutral facial expressions [*F*(1,37) = 163.11, *p* < 0.01, $\eta_{p}^{2}$ = 0.94], but ratings between the two races were not significant [*F*(1,37) = 1.90, *p* > 0.1], suggesting that the Chinese and Caucasian models’ facial expressions of pain and neutral facial expressions were comparable.

**Table 1:** Mean scores (standard deviation) for the faces in Experiment 1.

	Self-unpleasantness		Arousal		Likeability	
			
	Neutral	Pain	Neutral	Pain	Neutral	Pain
Caucasian face	2.34 (1.04)	5.61 (1.91)	4.38 (0.80)	6.63 (1.10)	5.16 (0.53)	3.15 (0.87)
Chinese face	2.29 (1.03)	5.61 (1.90)	4.37 (0.77)	6.66 (0.88)	5.00 (0.53)	3.11 (0.76)


The participants were told to perform two ordinal temporal bisection tasks (Chinese faces vs. Caucasian faces). The order of these two tasks was counterbalanced across participants. Each bisection task consisted of a learning phase that used 400 ms and 1600 ms as the “short” and “long” standard durations, respectively; two training phases were employed, one presenting the anchor durations and the other using six compared durations (400, 600, 800, 1200, 1400, and 1600 ms), as well as a test phase presenting all compared durations (400, 600, 800, 1000, 1200, 1400, and 1600 ms).

During the learning phase, participants needed to differentiate between and remember the “short” and “long” standard durations, with each duration presented randomly five times. In the first training phase, participants were instructed to answer whether the duration of the rectangle was “short” or “long” by pressing one of two keys (“D” and “K,” counterbalanced across participants). Feedback was given after the responses. This session consisted of three short and three long trials presented randomly. In the second training phase, participants needed to answer whether the duration presented was closer to the “short” or the “long” anchor. No feedback was given after the responses, but the accuracy rate was presented when the participants had completed all trials. If the accuracy rate was less than 70%, the participants underwent training again. In the test phase, the target stimuli were changed from the rectangle to faces, and the participants were informed. Each participant randomly completed 140 trials (10 models × 2 expressions × 7 durations) in each block.

The participants were told not to count the trials, as to prevent strategy effects that might bias the experimental data (Rattat and Droit-Volet, 2012).

### Results

**FIGURE 1:**
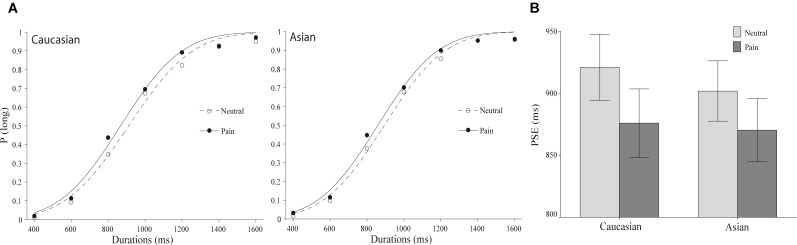
The results of Experiment 1. **(A)** Proportion of “long” responses plotted against durations ranging between 400 and 1600 ms for the painful and neutral expressions displayed by Caucasian (left panel) and Chinese (right panel) models. The lines are the best-fit cumulative Gaussian curves for the two parameters (mean and SD). **(B)** Mean point of subjective equality for Caucasian (other-race) and Chinese (same-race) pain and neutral expressions. Error bars represent standard errors.

As illustrated in Figure 1A, from left to right, the starting point of the sigmoid curve represent the proportion of “long” responses to the shortest duration, and the end of the curve indicates the proportion of “long” responses to the longest duration. These curves were produced by performing the cumulative Gaussian function on the proportion of “long” responses to each stimulus duration, under different conditions (expressions of pain and neutral expressions on Caucasian and Chinese faces).

The mean and SD of the regression curves are presented to compare the participants’ performance on the behavioral tests under each condition. The mean referred to the point of subjective equality (PSE; the duration that participants have a 50% chance of identifying it as a “long” response). A smaller PSE indicates that the participants make no distinction between “long” and “short” at a relatively short duration; therefore, the participants overestimate the duration. The SD corresponds to the WR. A larger WR value suggests a more gradual curve and lower sensitivity to temporal durations (Kroger-Costa et al., 2013; Liu et al., 2015; Tian et al., 2018).

The psychometric function for facial expressions of pain is clearly shifted to the left compared to the position of the psychometric function of neutral facial expressions for both other- and same-race facial expressions (Figure 1). This pattern reveals that stimulus durations were perceived as lasting longer when the participants viewed facial expressions of pain. Indeed, an overall ANOVA was conducted on the PSEs with facial expression (pain vs. neutral) and the models’ race (Chinese vs. Caucasian) as within-subjects variables and revealed a significant effect of facial expression, *F*(1,37) = 12.79, *p* < 0.01, $\eta_{p}^{2}$ = 0.26. The main effects of race and the expression × the race interaction were not significant, *F*(1,37) = 0.41 and 0.46, all *p* > 0.05.

An ANOVA was conducted on the WR. A significant effect was not observed [race, *F*(1,37) = 0.04, emotion, *F*(1,37) = 0.01, race × emotion, *F*(1,37) = 1.36, all *p* > 0.05], indicating that participants’ sensitivity to the temporal duration was not distorted by facial expressions of pain.

### Discussion

The aim of the present experiment was to explore whether facial expressions of pain influence temporal perception in a temporal bisection task and whether this effect was modulated by the racial group relationship. Participants were required to rate the presented facial expressions after a temporal bisection task. Facial expressions of pain were rated as more arousing, more negative and less likable than neutral facial expressions, but a significant difference was not observed between the facial expressions of same- and other-race members, suggesting that the ratings were comparable between Chinese and Caucasian faces.

No statistically significant effect on the WR was observed under all conditions, suggesting that facial expressions of pain do not impair sensitivity to temporal information, consistent with the results of previous studies indicating that emotional stimuli do not distort organisms’ temporal sensitivity (Gil and Droit-Volet, 2011; Fayolle et al., 2015).

Based on the results of the present experiment, the PSEs of facial expressions of pain were smaller than those of neutral facial expressions. The PSE results in the present study are consistent with the findings of previous studies using a similar range of durations (400–1600 ms), showing that subjective arousal and valence were closely related to the lengthening effect of the emotional stimulus on temporal perception (Ng et al., 2011; Smith et al., 2011; Gil and Droit-Volet, 2012). Same- and other-race facial expressions were comparable in these dimensions, indicating that they lengthen the perceived duration to a similar extent. Attractiveness did not explain the present results because face that were disliked, induced an underestimation rather than an overestimation, of the perceived duration compared to neutral faces (Ogden, 2013).

Importantly, we did not identify differences between the effects of same- and other-race facial expressions on the perceived duration. This finding refuted our hypothesis that facial expressions of pain of same-race members would produce a larger lengthening effect on the temporal perception than those of other-race members. However, this finding was consistent with the result of a previous study in which Chinese participants were presented with same- and other-race neutral and angry facial expressions (Mondillon et al., 2007).

In Experiment 2, we decided to use a short duration ranging between 200 and 800 ms to identify a multiplicative effect through a comparison with the results of Experiment 1 and to test whether the difference between same- and other-race facial expressions of pain, was not reflected by the temporal perception.

## Experiment 2

### Materials and Methods

#### Participants

Thirty-eight healthy undergraduate students (14 men and 24 women) from SWU, Chongqing, China, participated in the study. All participants were Chinese, aged between 17 and 29 years (mean = 20.2 years, *SD* = 1.27 years), right-handed, had normal/corrected-to-normal vision, and had no neurological problems. This study was approved by the SWU Research Ethics Committee. The participants were different from the individuals who completed Experiment 1.

#### Materials and Procedure

The procedure and the materials were similar to those adopted in Experiment 1, except that the anchor durations were replaced by 200 and 800 ms, and the probe durations were changed to 200, 300, 400, 500, 600, 700, and 800 ms.

### Results

**FIGURE 2:**
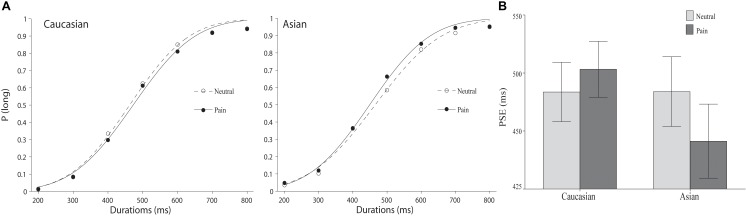
The results of Experiment 2. **(A)** Proportion of “long” responses plotted against durations ranging between 400 and 1600 ms for the pain and the neutral expressions displayed by Caucasian (left panel) and Chinese (right panel) models. The lines are the best-fit cumulative Gaussian curves for two parameters (mean and SD). **(B)** Mean point of subjective equality for Caucasian (other-race) and Chinese (same-race) pain and neutral expressions. Error bars represent standard error.

As illustrated in Figure 2, a leftward shift of the psychometric function for facial expressions of pain compared to the position of the psychometric function for neutral facial expressions was observed for same-race but not for other-race facial expressions. An overall ANOVA was conducted on the PSEs with expression (pain vs. neutral) and race (Chinese vs. Caucasian models) as within-subject variables. The main effects of facial expression and race were not significant, *F*(1,37) = 0.96 and 1.21, all *p* > 0.05, but a significant expression × race interaction was observed, *F*(1,37) = 12.60, *p* < 0.01, $\eta_{p}^{2}$ = 0.25. *Post hoc* paired samples *t*-tests confirmed that the PSE was shorter for pained facial expressions (*M* = 445.63, *SD* = 98.50) than for neutral facial expressions (*M* = 466.99, *SD* = 92.59) on Chinese faces, *t*(37) = 2.97, *p* < 0.01, but not Caucasian faces (pain: M = 476.61, *SD* = 74.50; neutral: M = 466.83, *SD* = 78.90), t(37) = 1.36, *p* > 0.05), suggesting that the stimulus durations were only perceived to be longer for pained facial expressions on Chinese faces than for the neutral facial expressions and not for Caucasian faces.

We calculated the difference in the PSEs between same-race facial expressions of pained and neutral facial expressions in Experiments 1 and 2 to test the hypothesis that the lengthened durations were caused by arousal, which would reveal a multiplicative effect. One-way ANOVA indicated a non-significant effect of duration, indicating that significant differences did not exist between the 400-1600 ms and the 200-800 ms durations, *F*(1, 74) = 0.44, *p* > 0.05. This result was not consistent with an interpretation confirming an arousal-based effect.

An ANOVA was conducted on the WR. No significant effect was found [race, *F*(1, 37) = 1.58, emotion, *F*(1, 37) = 0.82, race × emotion, *F*(1, 37) = 0.72, all *p* > 0.05], indicating that participants’ sensitivity to the temporal duration was not distorted by facial expressions of pain.

### Discussion

The aim of the present experiment was to identify a multiplicative effect and to test whether the effects of same- and other-facial expressions of pain, on temporal perception, differ at a relatively early stage. Although same- and other-race faces were rated similarly, the results of the temporal bisection task revealed that their impacts on temporal perception differed. Based on the results of the present experiment, the PSE for same-race facial expressions of pain was less than the PSE for same-race neutral facial expressions, suggesting that same-race facial expressions of pain lengthened participants’ perceived duration. The PSE for other-race facial expressions of pain was not significantly different from the PSE of other-race neutral facial expressions, suggesting that other-race facial expressions of pain did not lengthen the subjective duration.

Similar to the results of Experiment 1, a statistically significant effect of expressions of pain on the WR was not observed under all conditions. Combined with the result of WR in Experiment 1, facial expressions of pain do not impair sensitivity to time information across different durations.

According to the SET, arousal and attention should produce a multiplicative and additive effect on temporal perception, respectively. We compared the effects of facial expressions of pain to the results obtained in Experiments 1 and 2. For same-race faces, the magnitude of time distortion caused by facial expressions of pain did not increase at longer durations (400-1600 ms). Thus, these results do not appear to be consistent with the assumed arousal effect. In addition, for other-race faces, the result was more complicated; other-race faces lengthened the subjective duration at relatively long durations but not at relatively short durations. Thus, neither an additive nor a multiplicative effect was observed, but a multiplicative effect may exist if the lengthening effect on short duration ranges is too small to be mathematically significant. We will return to this conundrum in the general discussion.

## General Discussion

The study attempted to identify the influence of facial expressions of pain on temporal perception and whether this effect was modulated by the racial group relationship between an observer and a target. We employed a temporal bisection task and presented our Chinese participants with facial expressions of pain or neutral facial expressions displayed by Chinese (same race) or Caucasians (different race). These stimuli were rated by the participants after a bisection task, as described in Experiment 1. According to the rating scores, facial expressions of pain were more arousing, unpleasant and disagreeable than neutral facial expressions. Importantly, a significant difference was not observed between the facial expressions of same- and other-race members. While rated as comparable, these facial expressions have distinct impacts on temporal perception. Specifically, when we adopted 200 and 800 ms as standard durations, participants tended to overestimate the durations when presented with same-race facial expressions of pain compared to neutral facial expressions, but this tendency was not found for other-race facial expressions. However, when we used longer probe durations ranging from 400 to 1600 ms, other-race facial expressions of pain significantly lengthened perceived durations to a magnitude similar to same-race facial expressions of pain. A statistically significant effect of pained facial expressions on the WR was not observed at either the 200-800 ms or 400-1600 ms duration range, suggesting that pained facial expressions do not influence a person’s sensitivity to temporal information.

In line with the previous studies, the present study indicated that pain-related stimuli lengthened the perceived duration. Physically painful stimuli, such as electric shock and painfully cold water, have been reported to significantly extend temporal perception (Hare, 1963; Ogden et al., 2015; Rey et al., 2017). For example, using a modified verbal estimation paradigm in which the participants must temporally estimate three different shapes that indicate a forthcoming or occurring heat-induced pain or a control condition, participants overestimated the duration of shapes associated with pain (Ogden et al., 2015). Thus, in the present study, facial expressions of pain, similar to other physically painful stimuli, lengthened the perceived duration, supporting the hypothesis that the perceptions of others’ or one’s own pain share certain similarities with respect to emotion (Singer et al., 2004; Rütgen et al., 2015).

As shown in the present study, same-race facial expressions of pain produced an intercept effect on temporal perception. According to the internal clock model of the SET (Gibbon, 1977; Gibbon et al., 1984), the constant lengthening effect of same-race facial expressions of pain on time perception suggested an attention-switch mechanism. Indeed, facial expressions of pain induce early attention engagement and enhance sustained attention (Decety et al., 2010; Menon and Uddin, 2010; Baum et al., 2013), which may shorten the latency to the onset of the temporal process or help maintain temporal information to produce a longer perceived duration. However, arousing static images without any movement that change over time produce an additive effect on similar duration ranges and a multiplicative effect on short durations (100-800 ms), suggesting that the increase in arousal, associated with static images, is ephemeral and returns to the baseline after a few hundred milliseconds (Nather et al., 2011; Gil and Droit-Volet, 2012). This is in contrast with the effect of emotional films, which induce a much more persistent emotional effect on temporal perception (Fayolle et al., 2014). Previous studies have shown that physically painful stimuli induce a longer perceived duration via both attention and arousal mechanisms (Ogden et al., 2015; Rey et al., 2017). In summary, we speculate that facial expressions of pain lengthen the perceived durations via arousal and attention, and this effect is short-lived.

A short-lived lengthening effect of facial expressions of pain may help to explain the result of a previous study that also used facial expressions of pain as the stimuli but found an underestimation rather than overestimation of the perceived duration (Ballotta et al., 2018). The duration ranges we used here were different; the stimulus durations in our experiments were no longer than 1600 ms, while in the previously reported experiment, the participants were required to produce a subjective interval of 3 s after the presentation of the stimulus. Previous studies have asserted that the lengthening effect resulting from static emotional images is short-lived (Bar-Haim et al., 2010; Nather et al., 2011). Therefore, the temporal lengthening effect of facial expressions of pain may be attenuated and disappear after a few seconds, and other factors may contribute to a shortening effect. These factors may include attention, because pain-associated stimuli were attention-grabbing early in the stimulus presentation but gradually became unattended (Priebe et al., 2015).

Most importantly, the racial group relationship modulated the effect of facial expressions of pain on time perception in the present study. Indeed, other-race pained facial expressions affected temporal perception at relatively long durations (400-1600 ms) but did not exert a significant effect at relatively short durations (200-800 ms). Compared to the effect of same-race pained facial expressions, which produce a lengthening effect at both relatively short and long durations, the results for other-race facial expressions of pain, suggest that people need more time to perceive the pain of other-race individuals relative to same-race individuals, leading to a delayed lengthening effect on temporal perception. Indeed, the pained facial expressions of same-race members elicit larger P2-N2 amplitudes at approximately 180-300 ms, but these effects are not observed for other-race faces (Sheng and Han, 2012; Sheng et al., 2013; Huang and Han, 2014). Although perceived later, other-race individuals’ facial expressions of pain lengthen the perceived duration, supporting the assumption that the perception of the pain expressed by other-race faces is a slower and more controlled process than the perception of same-race faces (Sessa et al., 2014). The effect of other-race facial expressions of pain was likely very small (and thus not clearly significant) at relatively short durations but became larger (and significant) at relatively long durations. If this hypothesis is true, then a multiplicative effect occurred, consistent with the assumption of an arousal effect.

The different effects of same- and other-race pained facial expressions on temporal perception may stem from their different implications for an individual’s survival. As social mammals, the pain of in-group members is closely related to one’s own safety (Williams, 2002). The early perception of others’ pain helps an organism to efficiently adapt with heightened arousal and attention to actual or potential threats (Yamada and Decety, 2009; Ibáñez et al., 2011). Individuals must efficiently perceive the pain suffered by same-race individuals to execute an adaptive response, and thus the perception of same-race members’ pain become automatic, producing a lengthening effect of temporal perception at a relatively early stage and helping individuals make quick decisions during social interactions. However, an out-group members’ pain is not perceived automatically because it is not directly related to one’s own safety (Cikara et al., 2011). As the emotional temporal distortion reflects the adaptive function of temporal perception (Droit-Volet and Gil, 2009), the result of the present study, that same-race facial expressions of pain affect temporal perception earlier than other-race pain perceptions, supports the hypothesis that the pain expressed by same-race members has a higher adaptive value than pain expressed by other-race members (Cikara et al., 2011).

Our study had some limitations. First, we asked participants to rate their arousal level induced by the faces, which reflected their subjective arousal. Higher subjective arousal is usually related to physiological arousal, such as a greater increase in skin conductance (Rickard, 2004) or a higher heart rate (Pollatos et al., 2007). However, these types of arousal were shown to be modulated separately (Britton et al., 2006). Psychophysiological measurements of arousal and attention (e.g., event-related potentials, galvanic skin response and eye movements) might be useful to more directly assess early enhanced arousal and attention when participants are processing the facial pain expressions of same-race targets and a later enhancement for other-race targets, which was inferred from the present results. In addition, a previous study presented Chinese and Caucasian participants with angry and neutral faces displayed by same- and other-race models and found that same- and other-race angry faces lengthened the perceived duration of Chinese participants, but only same-race angry faces affected the Caucasian participants’ temporal perception. The authors attribute this difference to cultural differences of self-construction (Mondillon et al., 2007). Future studies should explore how these cultural differences modulate the effects of facial expressions of pain on same- and other-race faces on temporal perception.

In summary, the present study attempted to explore the effects of same- and other-race facial expressions of pain on temporal perception. Facial expressions of pain lengthened individuals’ subjective duration via arousal and attention without the distortion of sensitivity to temporal information. More importantly, individuals’ temporal perception was affected by same-race pained facial expressions at an early stage, but this effect occurred at a relatively late stage for other-race pained facial expressions, suggesting that individuals perceive the pain expressed by same-race members earlier than pain expressed by other-race members.

## Author Contributions

SH, JQ, QL, PL, and XH designed the experiments. SH acquired the data. SH and JQ analyzed the data. All authors contributed to the interpretation of the data and approved the final version of the manuscript. SH and JQ are co-first authors for the repeated discussions about the design, the statistical analysis of the results, the writing and arrangement of this paper, and the responses for the problems raised by reviewers.

## Conflict of Interest Statement

The authors declare that the research was conducted in the absence of any commercial or financial relationships that could be construed as a potential conflict of interest.
